# Periodontitis in Cardiovascular Disease Patients with or without Marfan Syndrome -A Possible Role of *Prevotella intermedia-*

**DOI:** 10.1371/journal.pone.0095521

**Published:** 2014-04-18

**Authors:** Jun-ichi Suzuki, Yasushi Imai, Mieko Aoki, Daishi Fujita, Norio Aoyama, Yuko Tada, Kouji Wakayama, Hiroshi Akazawa, Yuichi Izumi, Mitsuaki Isobe, Issei Komuro, Ryozo Nagai, Yasunobu Hirata

**Affiliations:** 1 Department of Advanced Clinical Science and Therapeutics, The University of Tokyo, Bunkyo, Tokyo, Japan; 2 Department of Cardiovascular Medicine, The University of Tokyo, Bunkyo, Tokyo, Japan; 3 Department of Periodontology, Tokyo Medical and Dental University, Bunkyo, Tokyo, Japan; 4 Department of Cardiovascular Medicine, Tokyo Medical and Dental University, Bunkyo, Tokyo, Japan; 5 Jichi Medical University, Shimotsuke, Tochigi, Japan; 6 Tokyo Teishin Hospital, Chiyoda, Tokyo, Japan; Glaxo Smith Kline, Denmark

## Abstract

**Background:**

Although periodontitis is a risk factor for cardiovascular disease (CVD), the influence of periodontitis on Marfan syndrome (MFS) with CVD is unclear. The aim of this study was to assess the relationship between periodontal bacterial burden and MSF with CVD.

**Methods and Results:**

The subjects were patients with MFS with CVD (n = 47); age and gender matched non-MFS CVD patients (n = 48) were employed as controls. Full-mouth clinical measurements, including number of teeth, probing of pocket depth (PD), bleeding on probing (BOP) and community periodontal index (CPI) were recorded. We also evaluated the existence of three periodontal pathogens, *Porphyromonas gingivalis*, *Aggregatibacter actinomycetemcomitans*, and *Prevotella intermedia* using polymerase chain reaction assays. Serum antibody titers against the pathogens were also measured. We revealed that MFS with CVD patients had periodontitis more frequently than the age and gender matched non-MFS CVD control subjects. MFS with CVD patients had significantly severer periodontitis, fewer remaining teeth and deeper PD compared to the non-MFS CVD controls. Furthermore, the serum antibody titer level against *Prevotella intermedia* was significantly lower in MFS plus CVD patients compared to the non-MFS CVD patients.

**Conclusion:**

Periodontitis may influence the pathophysiology of cardiovascular complications in MFS patients. A specific periodontal pathogen might be a crucial therapeutic target to prevent CVD development.

## Introduction

Marfan syndrome (MFS), which is a systemic connective tissue disorder, often shows complications of cardiovascular disease (CVD), such as aortic aneurysm, cardiac valve abnormality, and infective endocarditis [1]. It is well known that MFS frequently displays oral manifestations, such as local hypoplastic enamel spots, root deformity, abnormal pulp shape, pulpal inclusions, calculus and gingival indices [2]. Thus, individuals with MFS were at a high risk of bacteremia-induced CVD with dental disorders [3]. However, there has been no report to reveal the morbidity of periodontitis in Japanese MFS patients with CVD. The aim of this clinical study was to compare the prevalence of periodontitis and specific bacterial burden between MFS plus CVD and non-MFS CVD patients. We, for the first time, revealed that severe periodontitis was frequently observed in MFS plus CVD patients and that periodontal pathogen might affect CVD pathogenesis.

## Methods

### 1. Subjects

The subjects were MFS plus CVD patients (n = 47). MFS was diagnosed with clinical criteria (the revised Ghent nosology) [4]; CVD included aortic aneurismal (n = 43) and cardiac valvular (n = 18) disorders; some MSF patients had both diseases. Age and gender matched non-MFS CVD individuals (n = 48) were employed as a control group. The control CVD group included arrhythmia (n = 34), peripheral arterial disease (n = 7), cardiomyopathy (n = 5) and myocardial ischemia (n = 2). We compared the blood levels of C-reacting protein (CRP) and brain natriuretic peptide (BNP). The protocol of the present study was approved by the Ethics Committee of the Schools of Medicine, the University of Tokyo (approved number 3059) and Tokyo Medical and Dental University (approved number 1165). It was conducted in accordance with the Helsinki Declaration of 1975, as revised in 2000. Written informed consent was obtained from all participants.

### 2. Periodontal examination

Periodontal examinations were performed by dentists who were not familiar with the clinical systemic findings of these individuals. Their examinations were performed routinely and without bias. Full-mouth clinical measurements, including probing of pocket depth (PD), bleeding on probing (BOP) were recorded using a manual probe (PCP-UNC 15, Hu-Friedy Manufacturing Co., Chicago, IL, USA) at six points (buccal-mesial, mid-buccal, buccal-distal, lingual-mesial, mid-lingual, lingual-distal) on a right upper molar, an upper incisor, a left upper molar, a right lower molar, a lower incisor and a left lower molar. We did not examine the third molars because they were occasionally impacted. We also evaluated the number of remaining teeth and the community periodontal index (CPI, grade 0–4).

### 3. Real-time Polymerase Chain Reaction (PCR) to Detect Bacterial Existence

Unstimulated saliva and dental plaque collected by paper points of each subject were obtained. Bacterial DNA was extracted from 200 µl saliva using DNeasy Blood and Tissue kit (Qiagen, Tokyo, Japan) according to the manufacturer's instructions, and was stored at −30°C until analysis. Real-time PCR method was used to detect three periodontopathic bacteria, *Porphyromonas gingivalis (P. gingivalis)*, *Aggregatibacter actinomycetemcomitans (A. actinomycetemcomitans*) and *Prevotella intermedia (P. intermedia)*. Specific primers for each bacterium were used as previously described [5]; 5′-cttgacttcagtggcggcag-3′ and 5′-agggaagacggttttcacca-3′ for *P. gingivalis*; 5′-cttacctactcttgacatccgaa-3′ and 5′-atgcagcacctgtctcaaagc-3′ for *A. actinomycetemcomitans*; and 5′-aatacccgatgttgtccaca-3′ and 5′-ttagccggtccttattcgaa-3′ for *P. intermedia*. The real-time PCR was performed using Thermal Cycler Dice^R^ Real Time System (Takara Bio Co., Shiga, Japan). The reaction mixture for SYBR Green assay (25 µl) contained 12.5 µl of SYBR^R^ Premix EX Taq II (Takara Bio Co., Shiga, Japan), 1 µl of forward and reverse primer (10 µmol/l) and 2 µl of extracted DNA. Thermocycling program was 40 cycles of 95°C for 5 seconds and 60°C for 30 seconds with an initial cycle of 95°C for 30 seconds. At each cycle, accumulation of PCR products was detected by monitoring the increase in fluorescence of the reporter dye from dsDNA-binding SYBR Green. After the PCR, a dissociation curve (melting curve) was constructed in the range of 60°C to 95°C. We determined a negative and positive in PCR analysis as follows. When bacterial count was more than 200, we determined it was positive. When the count was 200 or less, it was negative.

### 4. Enzyme-linked Immunosorbent Assay (ELISA) to Measure Anti-bacterial Antibodies

Serum samples were analyzed for IgG antibody against cell surface antigens for the following three suspected periodontal pathogens: *P. gingivalis*, *A. actinomycetemcomitans* and *P. intermedia* using an enzyme-linked immunosorbent assay (ELISA) as previously described [6]. Briefly, the microtiter plates were coated with sonicated whole cell extracts of *P. gingivalis* ATCC 33277, *A. actinomycetemcomitans* ATCC 33384 and *P. intermedia* ATCC 25611. Following an overnight incubation at 4°C, the suspension was replaced with PBS containing 2% BSA, 5% sucrose and 0.1% NaN_3_ to block the reaction, followed by four-hour incubation at 37°C. The plate was then washed three times with PBS-T (1 x PBS, 0.05% Tween 20, pH 7.2). Aliquots of 1,000-fold diluted serum samples and six different concentrations of reference solution were added to each well and incubated for 2 hours at 37°C. The plate was then washed and incubated with a solution containing labeled anti-human IgG for 1 hour at 37°C. The plate was washed again, and 100 µl of substrate was added to each well. The reaction was allowed to develop at room temperature for 30 minutes and stopped by adding 100 µl of 2N sulfuric acid. The absorbance of each well was read using a microplate reader at 450 nm with a 650 nm reference wavelength. Individual serum antibody levels (Units/ml) were calculated from the standard curve obtained from the gradual dilutions of the reference.

### 5. Data Analysis

Numerical data were presented as means ± standard error of mean (SEM) and the differences were examined with Mann-Whitney's U test for two group comparisons. Chi-square test was performed to compare gender, numbers of patients with periodontitis (CPI grade 3 and 4) and bacterial existence. All statistical analyses were performed with the aid of statistical software (Prism 5, ver. 5.0a, GraphPad Software). Values of P<0.05 were considered significant.

## Results

### 1. Patient Characteristics, Blood Examination and Periodontal Conditions

The patients' characteristics and data of blood examination data are presented in the **Table 1**. There was no statistical difference in age, gender, CRP and BNP between the two groups. The periodontal conditions of these patients are shown in **Table 2**. We revealed that MFS with CVD patients had periodontitis (CPI grade 3 and 4) more frequently (82.6%) than the age and gender matched non-MFS CVD control subjects (39.6%, p<0.05). MFS with CVD patients had significantly severer periodontitis (average CPI, 2.85±0.11 vs. 2.04±0.17, p<0.05), fewer remaining teeth (26.8±0.4 vs. 28.3±0.4, p<0.05) and deeper PD (3.07±0.09 vs. 2.33±0.07, p<0.05) compared to the non-MFS CVD controls. BOP rate was comparable between the two groups.

**Table 1:** Patient Characteristics and Blood Examination.

<Patient Characteristics>	MFS with CVD	non-MSF CVD	
Subject Numbers	47	48	NS
Male/Female	29/18	29/19	NS
Age	35.2±1.8	33.5±0.9	

<Blood Examination>	MFS with CVD	non-MSF CVD	
CRP (mg/dl)	0.32±0.13	0.33±0.13	NS
BNP (pg/ml)	26.1±4.8	44.7±10.1	NS

MFS, Marfan syndrome; CVD, cardiovascular disease; NS, not significant; CRP, C-reacting protein (CRP); BNP, brain natriuretic peptide.

**Table 2:** Periodontal Conditions.

	MFS with CVD	non-MSF CVD	
CPI Grade 3 and 4 (%)	82.6	39.6	p<0.05
Average CPI	2.85±0.11	2.04±0.17	p<0.05
Number of Remaining Teeth	26.8±0.4	28.3±0.4	p<0.05
Pocket Depth (mm)	3.07±0.09	2.33±0.07	p<0.05
Bleeding on Probing (%)	13.9±1.5	16.4±2.8	NS

MFS, Marfan syndrome; CVD, cardiovascular disease; NS, not significant; CPI, community periodontal index.

### 2. Bacterial Existence in Oral Samples

We found that the existence of *P. gingivalis* in saliva or dental plaque was comparable between the two groups. Similarly, the existence of *A. actinomycetemcomitans* or *P. intermedia* in saliva or dental plaque was not statistically different between the two groups **(data not shown)**.

### 3. Anti-bacterial Antibodies in Serum

The anti-bacterial antibody titers in blood serum are shown in **Table 3**. The serum antibody titer levels against *P. intermedia* were significantly lower in the MFS with CVD group (170,800±19,790 units/ml) compared to the non-MSF CVD group (273,800±45,020 units/ml, p<0.05). However, the serum antibody titer levels against *P. gingivalis* and *A. actinomycetemcomitans* were comparable between the two groups.

**Table 3:** Anti-bacterial Antibody Titers in Serum.

	MFS with CVD	non-MSF CVD	
Anti P.g. antibody	56,420±21,100	59,190±14,060	NS
Anti A.a. antibody	41,640±10,790	46,220±21,460	NS
Anti P.i. antibody	170,800±19,790	273,800±45,020	p<0.05
	(units/ml)	(units/ml)	

MFS, Marfan syndrome; CVD, cardiovascular disease; NS, not significant; CPI, community periodontal index; P.g., *Porphyromonas gingivalis*; A.a., *Aggregatibacter actinomycetemcomitans*; P.i., *Prevotella intermedia*.

## Discussion

MFS is a heritable disorder of connective tissue that is caused by mutations in the extracellular matrix protein fibrillin-1. It is a common pleiotropic disease with wide clinical variability and it should be diagnosed with its criteria [4], [7]. These criteria have features in the ocular, skeletal, integumental, respiratory and cardiovascular systems. We recently showed that common atherogenic risks affected aortic dilation in MFS patients [8]. Although oral manifestations were not included its criteria, they were frequently observed in MFS patients [2]. It is well known that each CVD disease has a different pathogenesis [9]. While it was reported that periodontitis is frequently seen in MFS patients [10], no data was provided to show its pathogenesis on CVD in MFS patients. Because periodontal tissues express fibrillin-1, its mutation must play a pivotal role to alter teeth support and gingival blood flow. Thus, MFS patients may be susceptible to periodontpathic bacteria that invade from the periodontal area with a connective tissue abnormality. In this brief report, we revealed that MFS plus CVD patients had periodontitis more frequently than the age and gender matched non-MFS CVD control subjects. Furthermore, MFS plus CVD patients had significantly severer periodontitis compared to that of the controls. This frequent and progressive periodontitis may result in the development of systemic periodontopathic bacteremia which may influence the condition of CVD in MFS patients.

We also demonstrated that the serum levels of anti-*P. intermedia* antibodies in MFS with CVD patients were significantly lower than the control groups, while its existence in saliva and plaque was comparable between the two groups. Although this observation study could not reveal the exact pathophysiological role of *P. intermedia* in the progression of CVD in patients with or without MSF, we can speculate on some possibilities. It was reported that *P. intermedia* infection was associated with carotid arterial atherosclerosis [11]. Guan et al. showed that *P. intermedia* stimulates tissue-type plasminogen activator and plasminogen activator inhibitor-2 expression via multiple signaling pathways in human periodontal ligament cells [12]. Other studies also demonstrated that lipopolysaccharide from *P. intermedia* activated nitric oxide [13], interleukin-6 and nuclear factor-κB [14]. This data suggested that *P. intermedia* might play a role in the pathogenesis of systemic disease via thrombotic and/or inflammatory factors. Because the MFS plus CVM patients had lower serum antibody titers with comparable existence of *P. intermedia* in oral tissues, they might have a weak defense system against the pathogen. It is well known that the amount of antibody production is varied among individuals. Our results suggested that many MFS patients could not effectively produce anti-*P. intermedia* antibody, therefore failing to disinfect the pathogen. Thus, we speculate that systemic infection of *P. intermedia* may affect on CVD development in MFS patients in a condition of immature defense and/or removal system. Because this is not a random sample examination, we could not draw epidemiologic conclusions. Further investigation is needed to reveal the mechanism and epidemiologic conclusions.

In conclusion, periodontitis may influence the pathophysiology of cardiovascular complications in MFS patients. A specific periodontal pathogen might be a crucial therapeutic target to prevent CVD development.
